# Ampicillin-Improved Glucose Tolerance in Diet-Induced Obese C57BL/6NTac Mice Is Age Dependent

**DOI:** 10.1155/2013/319321

**Published:** 2013-11-27

**Authors:** I. Rune, C. H. F. Hansen, M. Ellekilde, D. S. Nielsen, K. Skovgaard, B. C. Rolin, J. Lykkesfeldt, K. Josefsen, B. Tranberg, P. Kihl, A. K. Hansen

**Affiliations:** ^1^Section of Experimental Animal Models, Department of Veterinary Disease Biology, Faculty of Health and Medical Sciences, University of Copenhagen, Thorvaldsensvej 57, 1870 Frederiksberg, Denmark; ^2^Department of Food Science, Faculty of Science, University of Copenhagen, 1958 Frederiksberg, Denmark; ^3^Innate Immunology Group, National Veterinary Institute, Technical University of Denmark, Bülowsvej 27, 1870 Frederiksberg, Denmark; ^4^Translational Pharmacology, Novo Nordisk A/S, 2760 Måløv, Denmark; ^5^The Bartholin Institute, Rigshospitalet Department 3733, Copenhagen Biocenter, Ole Maaløes Vej 5, 2200 Copenhagen, Denmark

## Abstract

Ampicillin has been shown to improve glucose tolerance in mice. We hypothesized that this effect is present only if treatment is initiated prior to weaning and that it disappears when treatment is terminated. High-fat fed C57BL/6NTac mice were divided into groups that received Ampicillin at different ages or not at all. We found that both diet and Ampicillin significantly changed the gut microbiota composition in the animals. Furthermore, there was a significant improvement in glucose tolerance in Ampicillin-treated, five-week-old mice compared to nontreated mice in the control group. At study termination, expressions of mRNA coding for tumor necrosis factor, serum amyloid A, and lactase were upregulated, while the expression of tumor necrosis factor (ligand) superfamily member 15 was downregulated in the ileum of Ampicillin-treated mice. Higher dendritic cell percentages were found systemically in high-fat diet mice, and a lower tolerogenic dendritic cell percentage was found both in relation to high-fat diet and late Ampicillin treatment. The results support our hypothesis that a “window” exists early in life in which an alteration of the gut microbiota affects glucose tolerance as well as development of gut immunity and that this window may disappear after weaning.

## 1. Introduction 

Type 2 diabetes (T2D) is an increasingly omnipresent disease not only in the western world but also in many of the fastest developing third world countries [1]. It is caused by peripheral insulin resistance and an insulin production unable to compensate [2]. During the past decade, gut microbiota composition has been in focus to unravel the enigma of such lifestyle diseases and their development [3]. In animal models, gut microbiota composition has been shown to influence the development of a variety of autoimmune and inflammatory diseases such as type 1 and type 2 diabetes, rheumatoid arthritis, atherosclerosis, inflammatory bowel disease, and a range of allergies [4].

Leptin-deficient obese (lep^ob^) mice that develop glucose intolerance have a significant reduction in Bacteroidetes and an increase in Firmicutes compared with their wild-type lean litter mates [5]. Furthermore, the obese phenotype from lep^ob^ mice may be transplanted with the gut microbiota to germ-free wild-type mice [6]. Diet-induced obese (DIO) mice also exhibit a modified composition of the gut microbiota, endotoxemia, and an increased intestinal permeability [7]. Mechanistic explanations are still somewhat theoretical, and theories range from decreased early priming of intestinal regulatory T cells (T_reg_) leading to inadequate suppression of T helper cells (T_h_) later in life—the so-called “Hygiene Hypothesis” [8]—to transfer lipopolysaccharides (LPS) over a leaky gut in sensitive individuals [9]. An essential role of the gut microbiota is to facilitate energy harvest from otherwise indigestible components in our diet. Therefore, it is reasonable to assume that the gut microbiota has an impact on gut lipid metabolism. The reconstitution of germ-free mice with a normal microbiota increases total body fat and leads to a greater capacity to harvest energy from the diet and decreased insulin sensitivity [10]. Germ-free mice compared with conventional mice show decreased lipogenic-related gene expression [11]. However, several studies indicate that mechanisms are more sophisticated than simply being linked up to gut lipid metabolism. It has been hypothesized that peripheral insulin resistance is augmented by stimulation of intestinal Toll-like receptor 4 (TLR4) primarily by LPS from Gram-negative Proteobacteria leading to secretion of proinflammatory cytokines such as tumor necrosis factor alpha (TNF_*α*_). This has been exemplified by continuous subcutaneous infusion of LPS in mice, which increases glycemia and insulinemia and resulted in weight gain of liver, adipose tissue, and whole-body [12]. Alternatively, peptidoglycan from Gram-positive bacteria stimulates TLR2 and activates innate immunity [13], and therefore the lack of such stimulation may be expected to increase low-grade inflammation due to the lack of regulatory immunity. Ampicillin is a broad-spectrum antibiotic which can be used to target both Gram-positive and Gram-negative bacteria. Ampicillin treatment for longer periods, such as two-three weeks in lep^ob^ mice [14], four weeks in wild-type nonmodified C57BL/6 mice [15], and eight weeks in DIO Swiss mice [16], improves glucose tolerance, whereas the narrow-spectrum antibiotic erythromycin targeting mainly Gram-positive bacteria does not seem to have any effect [15]. Consequently, it is more likely that low-grade inflammation causing glucose intolerance is correlated to Gram-negative bacteria and subsequent LPS and TLR4 stimulation rather than to Gram-positive bacteria and subsequent TLR2 stimulation. This is also supported by the fact that TLR4 deficient mice are resistant to the induction of glucose intolerance through a high-fat diet (HFD) [17]. The impact on glucose tolerance in Ampicillin-treated wild-type C57BL/6 mice is not combined with impact on growth or gut regulatory immunology [15], whereas in the DIO Swiss mice, Ampicillin in addition to improving glucose tolerance also reduces the levels of insulin, TNF-*α*, IL-6, and TLR4 activity [16]. The difference between these two studies may be that low-grade inflammation is actually not induced in wild type C57BL/6 mice, which are known to develop impaired glucose tolerance spontaneously [18], whereas HFD in mice, as it has been used in Swiss mice [16], is known to induce a low-grade inflammation [19]. Therefore, there might be a higher number of immune-active cells to impact on Ampicillin treatment in HFD mice.

The mechanism behind improved glucose tolerance due to broad-spectrum antibiotic treatment may simply be related to a reduced transfer of LPS over an immature and permeable gut. However, the gut may not be equally permeable at any time of age [9]. All previous studies have initiated Ampicillin treatment early in life and continued it throughout the study [14–16]. The preweaned gut seems to be more permeable than the weaned gut [20, 21], and therefore glucose tolerance may only be induced with antibiotics if initiated early in life. Furthermore, if the gut microbial impact on glucose tolerance to a wide extent should be linked to the transfer of LPS from gut to serum, the intolerance would return rather quickly after terminating the antibiotic treatment, and there would be no lasting effects to reveal in the immune system. Consequently, the aim of this study was to determine whether a specific time frame exists in which manipulation of the gut microbiota by means of broad-spectrum antibiotic treatment would have an impact on disease development, here shown as glucose tolerance, and whether early intervention would have a lasting effect.

## 2. Results 

### 2.1. Animal Weights

At five weeks of age, no weight differences could be demonstrated between the groups (Figure 2(b)). At 11 weeks of age, both HFD-fed groups of animals were significantly heavier than their LFD counterparts, whereas no difference could be demonstrated between the two HFD groups. At 16 weeks of age, a difference was found only between the nontreated HFD group and the LFD group (Figure 2(b)).

### 2.2. Glucose and Insulin

At five weeks of age, a significant increase was found in oral glucose tolerance in Ampicillin-treated HFD mice (Group 1; Ampicillin+/DIO+) compared with nontreated HFD mice (Group 2; Ampicillin-/DIO+) (AUC, *P* = 0.0067; Figure 2(a)). However, at 11 weeks of age, that is, six weeks after terminating the Ampicillin treatment, the glucose tolerance in the Ampicillin-treated HFD group (Group 1; Ampicillin+/DIO+) was significantly lower compared to the low-fat diet (LFD) control animals (Group 3; Ampicillin−/DIO−) (*P* = 0.04; Figure 2(a)). Ampicillin treatment for four weeks from 12 to 16 weeks of age did not cause any differences in oral glucose tolerance, but the HFD mice treated with Ampicillin at an early age (Group 1A; Ampicillin 5 w+ 16 w−/DIO+) were still significantly less glucose tolerant than low-fat fed mice (Group 3; Ampicillin 5 w− 16 w−/DIO−) (*P* = 0.028; Figure 2(a)). Fasting insulin levels were not significantly different between any group at any point of measurement during the study. At six weeks of age, the glycated hemoglobin (HbA1c) values of the Ampicillin-treated HFD mice (Group 1; Ampicillin+/DIO+) were significantly lower than the values of the nontreated HFD mice (Group 3; Ampicillin−/DIO−) (*P* = 0.037; Figure 2(c)), and this was still the case at 17 weeks of age for the HFD mice treated with Ampicillin in early life (*P* = 0.036 for the mice treated once (Group 1A; Ampicillin 5 w+ 16 w−/DIO+), and *P* = 0.029 for those treated twice (Group 1B; Ampicillin 5w+ 16w+/DIO+)).

### 2.3. Plasma Cytokines and Lipopolysaccharides (LPS)

In the mice that were not treated with Ampicillin at any time, IL-6 was significantly lower in HFD mice compared to the values of LFD mice at 17 weeks of age (*P* = 0.039). No other differences were found in plasma cytokines measured at study termination (Figure 3; Table 1). TNF-*α* levels were measured, but all measurements were below detection limit.

LPS levels were measured at six weeks of age and again at 17 weeks of age. At no point in time significant differences between any of the groups (Figure 4) were found.

### 2.4. Gut Microbiota

Cluster analysis of denaturing gradient gel electrophoresis (DGGE) profiles obtained at five weeks of age showed a similarity of 0% when comparing all animals. At 11 weeks of age the overall similarity was 41%, and at 16 weeks of age the overall similarity was 23%. When comparing different points in time for nontreated animals throughout the study, a similarity of 43% was obtained for both HFD and LFD mice.

Analysis of entry coordinates obtained from Principal Component Analysis (PCA) plots showed a significant difference in gut microbiota at five weeks of age in relation to both Ampicillin treatment (PC1: *P* = 0.000, PC2: *P* = 0.001; Figure 5(a)) and diet (PC1: *P* = 0.000, PC3: *P* = 0.05; Figure 5(c)), and this was also the case at 16 weeks of age (Ampicillin PC1: *P* = 0.000; Figure 5(b)) (Diet PC1: *P* = 0.000; Figure 5(d)), whereas no differences could be demonstrated during the period of no Ampicillin treatment, that is, at 11 weeks of age, except for a borderline difference in relation to diet (PC2: *P* = 0.060).

### 2.5. Expression Analysis in Ileum

The expression of the mRNA of both serum amyloid A (SAA) (*P* = 0.0012) and interleukin 18 (IL-18) (*P* = 0.0014) was downregulated in HFD mice (Figure 6(a)). Although some variation was seen between the animals, both SAAmRNA (*P* = 0.032) and TNFmRNA (*P* = 0.029) were found to be expressed two to 15 times more in mice treated with Ampicillin at five and 16 weeks of age compared to mice only treated late (Figure 6(b)). Tumor necrosis ligand superfamily 15 (TNFSF15) mRNA was found to be significantly downregulated in the mice treated with Ampicillin at five and 16 weeks of age compared to those only treated in late life (*P* = 0.002; Figure 6(b)). Lactase was upregulated threefold in the mice only treated with Ampicillin early in life compared to the all other groups of HFD mice, but the difference was only found to be significant compared to those treated with Ampicillin twice (*P* = 0.044; Figure 6(b)).

### 2.6. Flow Cytometric Analyses of Dendritic Cells and Regulatory T Cells

Higher percentages of CD11b positive dendritic cells (CD11c+) were found systemically in the spleen from HFD mice compared to the LFD mice (Figure 7(a)), whereas a decrease in splenic dendritic cells expressing the tolerogenic marker CD103 was seen in the same mice (Figure 7(d)). Furthermore, both groups of HFD mice treated with Ampicillin later in life were found to have a lower amount of tolerogenic dendritic cells compared to the other groups independent of an early life Ampicillin treatment (Figure 7(e)). No significant differences were found in the Peyer's patches, and no other differences were detected among the dendritic and regulatory T cells (FoxP3 positive) (Figure 7).

## 3. Discussion 

Initiation of Ampicillin treatment from birth had a clearly beneficial effect on glucose tolerance which was not the case when these early treated mice were tested later in life, although at 17 weeks of age their HbA1c was still lower. HbA1c reflects long-term blood glucose and may at 17 weeks of age still be under impact of the early life Ampicillin treatment. It is also interesting to note that during the remaining part of the study, the early treated mice were significantly less glucose tolerant than the control group, which supports a newly published observation that subtherapeutic antibiotic therapy increased adiposity in young mice [22].

Three genes involved in inflammatory responses, namely, TNF, TNFSF15, and SAA, were found to be differentially expressed according to the time of Ampicillin treatment. In mice the gene SAA2, which in this study was genetically upregulated in treated mice, is expressed and induced principally in the liver by the proinflammatory cytokines IL-1, IL-6, and TNF-*α*. Hepatic SAA1 and SAA2 are induced up to a thousandfold in mice under acute inflammatory conditions following exposure to LPS [23]. Also extra hepatic expression of SAA in response to infection and inflammation in pig and cattle has been reported [24, 25]. This, along with TNFmRNA also being upregulated, supports that Ampicillin treatment, although inducing an acute improvement in glucose tolerance during treatment, actually leads to increased inflammation and a subsequent risk of reduced glucose tolerance after termination. However, in this study serum levels of IL-6 and IL-1 did not differ in mice treated in early life with Ampicillin compared to other mice on the HFD.

It could be hypothesized that Ampicillin treatment in this crucial phase of developing regulatory immunity interferes with the development of oral tolerance and therefore increases the risk of an inflammatory response when the gut bacteria reappear. Obesity in humans and HFD mice has previously been linked to depletion of regulatory T cells [26]. However, in another study, Ampicillin treatment did not seem to have a major impact on the regulatory T or NK cell related immunity [16]. Our study does not indicate that regulatory T or dendritic cell compartments, neither locally nor systemically, seem to play an important role in the altered glucose tolerance induced by the early life Ampicillin treatment. However, a shift in the ratio of CD11b positive and CD103 positive dendritic cells in HFD mice indicates that these may be of significance to the glucose intolerance induced by HFD, whereas this does not seem to be the case for regulatory T cells, at least not when monitored at 17 weeks of age. TNFSF15mRNA was significantly downregulated in the ileum of mice treated twice with Ampicillin compared to being treated only once. TNFSF15, also known as TL1A, is a potential vascular endothelial cell growth inhibitor [27], and it is related to inflammatory diseases of the gut, such as inflammatory bowel disease (IBD) [28] and irritable bowel syndrome (IBS) [29]. The expression of this inhibitor is regulated by several members of the gut microbiota [30], and therefore it is interesting, but not that surprising, that it is downregulated in the animals receiving the most intense Ampicillin treatment. Our observation that early life Ampicillin treatment upregulates lactase in the gut is in accordance with a study in which lactase also was upregulated in piglets raised under germ-free conditions compared to conventional piglets [31].

Ampicillin treatment clearly modified the gut microbiota at both points in time of treatment, but these gut microbiota changes did not seem to be lasting as there were no differences between the mice in the Ampicillin-free period.

The findings in the present study may speak in the favour of the theory that LPS during early life diffuses over a permeable mucosal barrier into the lamina propria and serum and thereby induces a low-grade inflammation through TNF-*α*. The lack of impact on serum TNF-*α*, which we observed, may seem to speak against this, but this was monitored at the end of the study and not during Ampicillin treatment. Preweaning reduction of the levels of gut LPS may, therefore, at this age improve glucose tolerance, while LPS diffusion may decrease after weaning due to decreased gut permeability [21]. In aged rats the permeability has then been shown to increase again [32]. Consequently, our study only gives some indication of the impact on pre-weaned and juvenile animals. It is also of importance that the impact of the HFD on glucose tolerance seems to decline during the study, which may, on the one hand, support the theory of LPS diffusion in early life as an essential factor but on the other hand leave less intolerance to be corrected by any experimental treatment. C57BL/6 mice have a high insulin secretory capacity and with age they will increase this to reduce the impact of peripheral low-grade inflammation on glucose intolerance [33]. This may also have been the case in this study. The fact that glucose tolerance in general seems to be lower at five weeks of age in this study may be due to the very young age of the animals at this point in time. It is recognised that stress due to handling of the animals may result in increased blood glucose levels.

IL-18 is known to induce IFN-*γ* production in natural killer (NK) cells and certain T cells as a response to LPS [34]. The HFD significantly downregulated mRNA coding for SAA and IL-18 in the gut. This is surprising because SAA is normally related to acute inflammation and the transport of cholesterol to the liver, where it also plays a role in various inflammatory diseases, such as atherosclerosis, and rheumatoid arthritis [23]. On the other hand, SAA is known to respond rapidly in the acute phase of inflammation, and it may be a compensatory effect that it is downregulated in the ileum if upregulated elsewhere in the organism over a longer period of time. The dietary impact on SAA corresponds to the observation that its inducer IL-6 was also significantly lower in plasma of the mice on the high-fat diet. The fact that IL-6 was higher in the low-fat fed animals compared to the high-fat fed animals was an unexpected finding as obesity and type 2 diabetes are connected to an increase of low-grade inflammatory cytokine such as IL-6.

To further study the impact of Ampicillin treatment on glucose tolerance, it would be valuable in future studies also to collect immunological data in the young mice during treatment, although this obviously calls for another experimental design in which animals are killed for gut sampling during the course of the study. It would also be of importance to correlate the level of gut permeability to the level of glucose intolerance, and it would be of interest to study even older animals.

In conclusion, changing glucose tolerance by means of antibiotic treatment in mice seems primarily possible in the very early life, and the improvement in tolerance disappears when treatment is terminated.

## 4. Materials and Methods 

### 4.1. Animals

Experiments were carried out in accordance with the European Union directive 86/609 on the Protection of Vertebrate Animals used for Experimental and Other Scientific Purposes, and the Danish Animal Experimentation Act number 1306 from November 23, 2007 which follows principles similar to “Principles of laboratory animal care” (NIH publication no. 85–23, revised 1985; http://grants1.nih.gov/grants/olaw/references/phspol.htm). The study was approved by the Animal Experiments Inspectorate, Ministry of Justice, Denmark.

Twenty-five presumed pregnant female C57BL/6NTac mice (Taconic Europe A/S, Ejby, Denmark) were divided into three groups. The pregnant mice gave birth to 40 male pups, which were individually earmarked (number 1–40) and randomized into cages with two to four animals in each group. The study continued for a total of 17 weeks counting from birth of the male pups. The animals were weighed once weekly from weaning. Prior to being killed by cervical dislocation at 17 weeks of age the animals were anaesthetized with Hypnorm/Dormicum mixture (VetPharm Ltd., Sherburn in Elmet, Leeds, UK; Roche A/S, Hvidovre, Denmark) (0.2 mL SC in a 1 : 1 : 2 water solution). The animals were daily subjected to visual control, and by signs of illness or misthriving the affiliated veterinarian was consulted.

### 4.2. Diets

**Figure 1:**
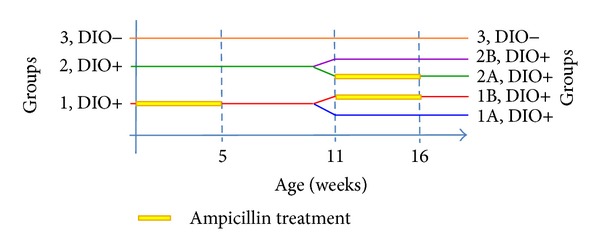
Experimental design for diet-induced obesity (DIO) and Ampicillin treatment in C57BL/6 mice.

**Figure 2:**
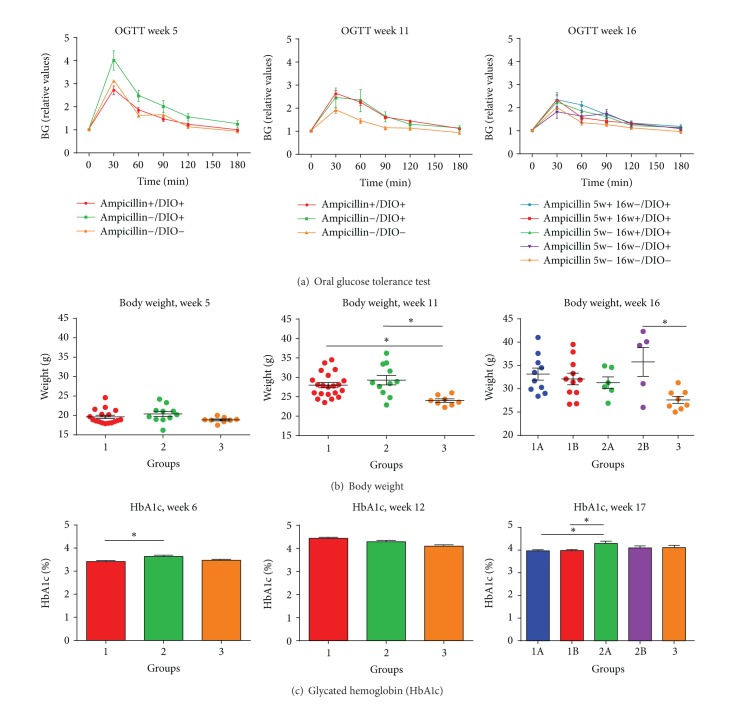
(a) Oral glucose tolerance (mean and SEM, relative values). Statistics were calculated on areas under the curves (AUC) and showed statistically significant differences between groups 1 (Ampicillin+/DIO+) and 2 (Ampicillin−/DIO+) at five weeks of age (*P* = 0.0067). At 11 weeks of age, no difference between the high-fat fed groups could be demonstrated, but differences between groups 1 (Ampicillin+/DIO+) and 3 (Ampicillin−/DIO−) were now evident (*P* = 0.04). At 16 weeks of age, differences between the early Ampicillin-treated group 1A (Ampicillin 5w+ 16w−/DIO+) and the low-fat fed group 3 (Ampicillin 5w− 16w−/DIO−) were still evident (*P* = 0.028). (b) At five weeks of age, no difference between body weights could be demonstrated, whereas differences between the high-fat fed groups 1 (Ampicillin+/DIO+) and 2 (Ampicillin−/DIO+) compared to the low-fat fed group 3 (Ampicillin−/DIO−) were evident at 11 weeks of age (*P* = 0.0028). At 16 weeks of age, only a difference between the nontreated high-fat fed group 2B (Ampicillin 5w− 16w−/DIO+) and low-fat fed group 3 (Ampicillin 5w- 16w−/DIO−) could be demonstrated (*P* = 0.0159) (mean and SEM depicted). (c) Glycated hemoglobin (% HbA1c, mean and SEM) shows differences between the high-fat fed groups 1 (Ampicillin+/DIO+) and 2 (Ampicillin−/DIO+) at six weeks of age (*P* = 0.037), whereas now difference could be demonstrated at 12 weeks of age. At 17 weeks of age, differences were found between the early treated groups 1A (Ampicillin 5w+ 16w−/DIO+) and 1B (Ampicillin 5w+ 16w+/DIO+) and the late treated group 2A (Ampicillin 5w− 16w+/DIO+), respectively (*P* = 0.036; *P* = 0.029).

**Figure 3:**
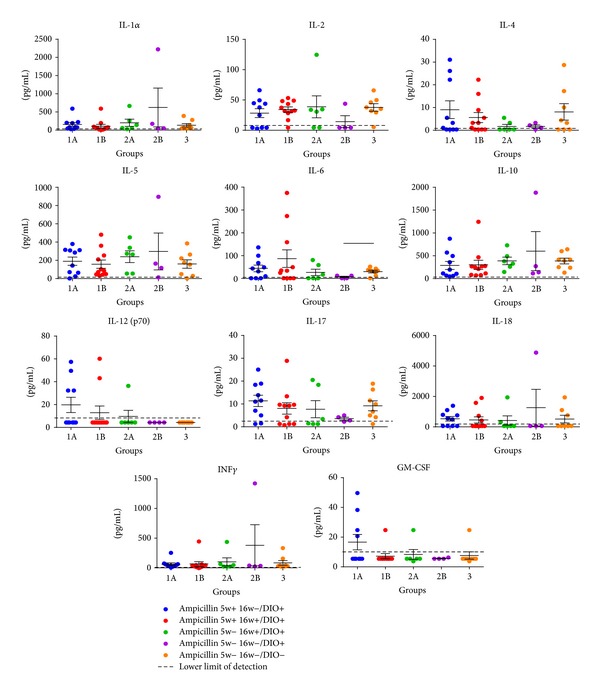
Levels of various plasma cytokines measured at week 17 at termination of the study (mean and SEM). IL-6 was statistically significantly higher in the low-fat fed group 3 (Ampicillin 5w− 16w−/DIO−) compared to the nontreated high-fat fed group 2B (Ampicillin 5w− 16w−/DIO+) (*P* = 0.039). TNF*α* levels (not shown) were also measured, but all measurements were under detection sensitivity.

**Figure 4:**
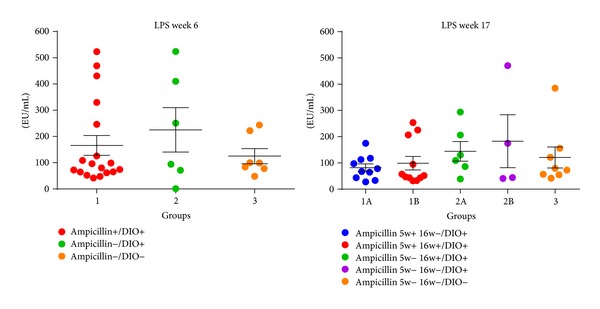
Lipopolysaccharide (LPS, mean, and SEM) levels in plasma were measured at six weeks of age and again at 17 weeks of age. At no point in time a statistically significant difference was present between the groups.

**Figure 5:**
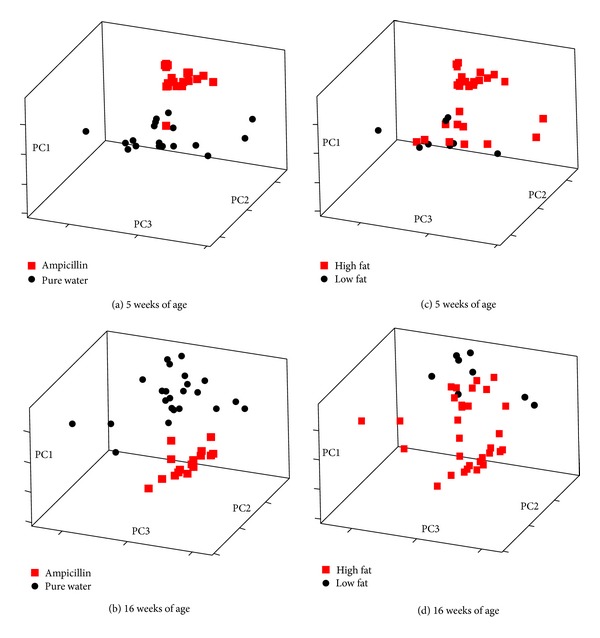
Principal component analysis (PCA) plots of the gut microbiota in diet-induced obese C57BL/6 mice treated or nontreated with Ampicillin from birth and until five weeks of age, from 12 to 16 weeks of age, or both. The clustering on all four PCA plots is significant: (a) PC1: *P* = 0.000, PC2: *P* = 0.001, (b) PC1: *P* = 0.000, (c) PC1: *P* = 0.000, PC3: *P* = 0.05, (d) PC1: *P* = 0.000.

**Figure 6:**
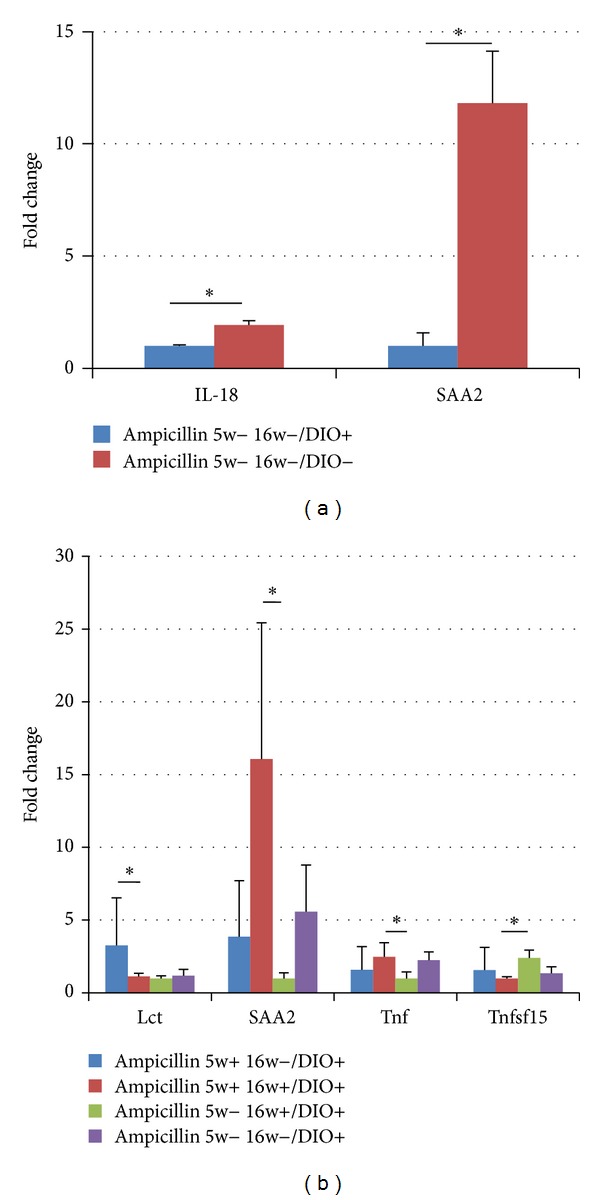
Significant differences in ileum mRNA expression as revealed by qPCR in diet-induced obese C57BL/6 mice treated or not treated with Ampicillin from birth and until five weeks of age (1A; Ampicillin 5w+ 16w−/DIO+), from 12 to 16 weeks of age (2A; Ampicillin 5w− 16w+/DIO+), at both points in time (1B; Ampicillin 5w+ 16w+/DIO+), or not at all (2B; Ampicillin 5w− 16w−/DIO+), as well as low-fat fed control mice (3; Ampicillin 5w− 16w−/DIO−). Interleukin 18 (IL 18), Lactase (Lct), serum amyloid A (Saa2), tumor necrosis factor (Tnf), Tumor necrosis factor ligand superfamily 15 (Tnfsf15). (a): SAA (*P* = 0.0012), IL-18 (*P* = 0.0014). (b): Lct (*P* = 0.044), Tnf (*P* = 0.029), SAA (*P* = 0.032), **Tnfsf15 (*P* = 0.002).

**Figure 7:**
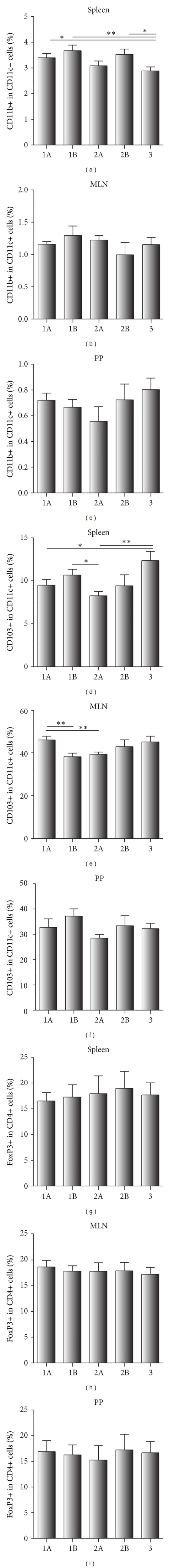
Flow cytometric analyses of lymphocytes isolated from the spleen, mesenteric lymph nodes (MLN), and Peyer's patches (PP). ((a)–(c)) Percentages of CD11b positive dendritic cells (CD11c^+^). ((d)–(f)) Percentages of tolerogenic CD103 positive dendritic cells. ((g)–(i)) Percentages of FoxP3 positive regulatory T cells (CD4^+^). High-fat diet (HFD) induced C57BL/6 mice treated with Ampicillin from birth and until five weeks of age (1A; Ampicillin 5w+ 16w−/DIO+), or from birth and until five weeks of age followed by Ampicillin treatment from 12 to 16 weeks of age (1B; Ampicillin 5w+ 16w+/DIO+), or only from 12 to 16 weeks of age (2A; Ampicillin 5w− 16w+/DIO+) are illustrated together with untreated HFD induced mice (2B; Ampicillin 5w− 16w−/DIO+) and untreated control mice (3; Ampicillin 5w− 16w−/DIO−). Error bars represent the SEM. *(*P* < 0.05), **(*P* < 0.01).

The animals in groups 1 and 2 were fed a high-fat diet (HFD) throughout the study (60% energy from fat, D12492, Research Diets Inc., New Brunswick, NJ, USA), whereas the animals in group 3 acted as a low-fat control group receiving a low-fat diet (LFD) throughout the study (10% energy from fat, C12450B, Research Diets Inc., New Brunswick, NJ, USA) (Figure 1). The feed was weighed and changed twice weekly.

### 4.3. Antibiotic Treatment

The animals in group 1 (*n* = 21) received the broad-spectrum antibiotic Ampicillin in their drinking water (1 g/L) (Ampivet vet., Boehringer Ingelheim, Copenhagen, Denmark) from three days prior to birth of the pups until the pups reached five weeks of age. The animals in groups 2 and 3 received pure drinking water (tap water) during this period (group 2 (*n* = 11), group 3 (*n* = 8)). From five weeks of age all animals received pure drinking water until week 12, where group 1 was subdivided into groups 1A (*n* = 10) and 1B (*n* = 11), and group 2 was subdivided into groups 2A (*n* = 6) and 2B (*n* = 5). The animals in groups 1B and 2A were shifted to water containing Ampicillin for the rest of the study. Groups 2B and 3 (*n* = 8) acted as HFD and LFD control groups, respectively, and received pure drinking water throughout the study. Water was changed twice weekly during periods of antibiotic treatment and once weekly during periods with no antibiotic treatment (Figure 1).

### 4.4. Glucose, Insulin, and HbA1c

Oral glucose tolerance test (OGTT) was performed at the end of the first treatment period (week 5), prior to the second treatment period (week 11) and at the end of the second treatment period (week 16). The mice were fasted overnight for 10 hours prior to the procedure. A baseline blood glucose level (*t* = 0) was measured by a Freestyle Mini Glucometer (Hermedico, Copenhagen, Denmark), and the mouse was immediately after gavaged with a glucose solution according to weight (Amgro I/S, Copenhagen, Denmark, concentration 500 g/l., dose 4 mL/kg). Blood glucose was then measured at *t* = 30, 60, 90, 120, and 180 min after gavage.

At weeks 6, 12, and 17 mouse plasma samples were analysed for insulin content using the Ultra sensitive Rat Insulin ELISA Kit (Crystal Chem, Downer's Grove, USA) with the modifications that sample volume was reduced to 5 *μ*L and that in-house rat insulin standards, prepared using heat-treated rat plasma, were used. Glycated hemoglobin (HbA1c) was measured on a Siemens DCA Vantage Analyzer (Siemens Healthcare Diagnostics, Ballerup, Denmark) by collection of 1 *μ*L full blood from a puncture in the tail vein in the supplied collection cassette.

### 4.5. Plasma Cytokines and Lipopolysaccharides (LPS)

The plasma cytokines IL-1*α*, IL-2, IL-4, IL-5, IL-6, IL-10, IL-17, TNF*α*, INF*γ*, and GM-CSF were measured by means of the Mouse Th1/Th2 10plex FlowCytomix Multiplex kit (Bender MedSystems, Vienna, Austria) in combination with two simplex kits; Mouse IL-12 (p70) FlowCytomix Simplex and IL-18 FlowCytomix Simplex (both Bender MedSystems). The assay was performed according to manufacturer's instructions. The analysis was run on a BD FacsCanto Flow Cytometer (BD Biosciences, Albertslund, Denmark) and processing of data was performed using the FlowCytomixTM Pro 2.3 Software (Bender MedSystems).

Plasma contents of LPS were measured using the PyroGene Recombinant Factor C Endotoxin Detection System (Lonza, Basel, Switzerland). The test utilizes recombinant factor C (rFC) which is an endotoxin-sensitive protein in combination with a fluorogenic substrate. The assay was performed according to manufacturer's instructions and fluorescence was measured before and after one-hour incubation at 37°C on a SpectraMax Plus 384 plate reader (Molecular Devices Inc., CA, USA).

### 4.6. Gut Microbiota

Fecal samples obtained aseptically at five, 11, and 16 weeks of age were analysed by means of DGGE as previously described [35]. In brief, bacterial DNA was extracted using the QIAamp DNA Stool Mini Kit (Qiagen, Hilden, Germany). Samples were homogenized prior to extraction using a FastPrep FP120 Cell Disrupter (QBiogene, MP Biomedicals, France) for 45 sec at 6 m/sec. Quality and concentration of the extracted DNA were verified on a NanoDrop 1000 Spectrophotometer (Thermo Scientific, USA). Genetic material was then amplified by Polymerase Chain Reaction (PCR), using primers specific to the V3 region of the 16S rRNA gene. Subsequently, genetic material was separated by means of DGGE on a polyacrylamide gel containing a 30%–65% chemical gradient (100% corresponds to 7 M urea and 40% formamide). DGGE profiles were analysed using BioNumerics version 4.5 (Applied Maths, Sint-Martens-Latem, Belgium) for cluster analysis (dice similarity coefficient with a band position tolerance and optimization of 1% using the unweighted pair Group method with arithmetic averages clustering algorithm (UPGMA)) and principal component analysis (PCA).

### 4.7. Gene Expression in Ileum

The ileum was sampled and frozen with liquid nitrogen immediately after cervical dislocation. Approximately 20–30 mg of the frozen tissue was then homogenized in 1 mL QIAzol Lysis Reagent (Qiagen) using gentleMACS Dissociator (Milteny Biotec, GmbH, Germany). Total RNA was extracted using RNeasy lipid Tissue midi kit (Qiagen), and all samples were treated with RNase-free DNase (Qiagen) (manufacturer's instructions). RNA purity was assessed using UV absorption spectrums including OD 260/280 and OD 260/230 ratios on a NanoDrop ND-1000 spectrophotometer (Saveen and Werner AB, Limhamn, Sweden). RNA integrity (RIN), which was between 6.1 and 8 for all samples, was measured on an Agilent 2100 Bioanalyzer (Agilent Technologies, Nærum, Denmark) using the RNA 6000 Nano Kit. Extracted RNA was converted into cDNA by reverse transcription of 500 ng total RNA using the QuantiTECT Reverse Transcription kit (Qiagen) containing a mix of random primers and oligo-dT (manufacturer's instructions). Two separate cDNA reactions were performed for each sample. cDNA was diluted 1 : 6 in low EDTA TE-buffer (VWR-Bie & Berntsen) prior to preamplification, which was completed using TaqMan PreAmp Master Mix (Applied Biosystems, Foster City, CA). A 200 nM pooled primer mix was prepared combining each primer used in the present study. TaqMan PreAmp Master Mix (5 *μ*L) was mixed with 2.5 *μ*L 200 nM pooled primer mix and 2.5 *μ*L diluted cDNA and incubated at 95°C in 10 min, followed by 16 cycles of 95°C in 15 sec and 60°C in 4 min. Preamplified cDNA was diluted at least 1 : 4 in low EDTA TE-buffer (VWR). Quantitative PCR (qPCR) primers were provided using the DELTAgene assay design service (Fluidigm Corporation, San Francisco, CA, USA) (Table 2). All primers were designed over introns. Primer amplification efficiencies and dynamic range were acquired from standard curves constructed from dilution series of highly responding samples. qPCR was performed in the 48.48 Dynamic Array Integrated Fluidic Circuits (Fluidigm) combining 48 preamplified samples with 48 primer sets for 2304 simultaneous qPCR reactions as previously described [36]. qPCR was performed in the BioMark real-time PCR instrument (Fluidigm Corporation), and the following cycle parameter was used: 2 min at 50°C, 10 min at 95°C, followed by 35 cycles with denaturing for 15 sec. at 95°C and annealing/elongation for 1 min at 60°C. Melting curves were generated after each run to confirm a single PCR product (from 60°C to 95°C, increasing 1°C/3 sec). Reactions were performed in duplicates (cDNA replicates). No template controls (NTC) were included to indicate potential problems with nonspecific amplification or sample contaminations. Nonreverse transcriptase controls were included to assess potential DNA contamination. Relative concentrations of target mRNA were assigned using standard curves constructed from three separate dilution series of highly responding samples (cDNA dilution 1 : 3, 1 : 15, 1 : 75, 1 : 375, 1 : 1875, and 1 : 9375). Data were acquired using the Fluidigm Real-Time PCR Analysis software 3.0.2 (Fluidigm Corporation).

### 4.8. Flow Cytometry

Cells were isolated from spleen, mesenteric lymph node (MLN), and Peyer's patches (PP) by aseptically squeezing the fresh organs in PBS between two microscope slides and subsequently passing the suspension through a 70 *μ*m cell strainer. Cell suspensions were stored on ice at all times. Spleen cells were resuspended in red blood cell lysis (ACK) buffer (0.15 M NH_4_Cl, 10 mM KHCO_3_, 1 mM EDTA monosodium pH 7.3) and incubated for six min. Subsequently, cells were washed and resuspended in PBS. Cells were surface stained for dendritic cell and T-cell markers and with appropriate isotype control antibodies. All antibodies (anti-mouse CD4, CD11c, CD11b, CD103, FoxP3) were purchased from eBioscience (San Diego, CA, USA). For the regulatory T-cell staining the cell was subsequently fixed, permeabilized, and intracellular Foxp3 stained according to the manufacturer's protocol. Analysis was performed using an Accuri C6 flow cytometer (Accuri Cytometers Inc., Ann Arbor, MI, USA).

### 4.9. Statistics

Normality distribution of the data was measured with Anderson-Darling's normality test considering *P* values less than 0.05 significant (Minitab, Coventry, UK). Statistical analysis of OGTT was performed on area under the curve (AUC) using Statistica (Statsoft, Tulsa, OH, USA) and statistical significance evaluated by two-way repeated measures ANOVA followed by post hoc analysis using Student's *t*-test in case of significant effects. For analysis of insulin, HbA1c, plasma cytokines, LPS, and PCA data, GraphPad Prism version 5 (GraphPad Software, San Diego, CA, USA) was used and the statistical significance evaluated by one-way ANOVA (Kruskal-Wallis test on data that did not assume Gaussian distributions) and Student's *t*-test (Mann-Whitney test on data that did not assume Gaussian distributions). Values below detectable limits (insulin and cytokine measurement kits) were given the value of half of the lover limit of quantification (1/2 LLOQ). For analysis of data from expression analysis of the ileum, data preprocessing, normalisation, relative quantification, and statistics were performed using GenEx5 (MultiD, Göteborg, Sweden). Data was log_2_ transformed for approaching normal distribution prior to *t*-test (2-tailed, unpaired). Gene expression was considered to be significantly different if the *P* value < 0.05 and fold change >±2.0. For analysis of FACS data, ANOVA followed by *t*-test for significant differences between groups was applied (Minitab).

## Figures and Tables

**Table 1:** Serum cytokines in diet-induced obese C57BL/6 mice treated or not treated with Ampicillin from birth to five weeks of age, from 12 to 16 weeks of age, or both.

Group	Ampicillin		DIO	*N*	IL-1a			IL-2			IL-4		
	Early	Late			Median	Minimum	Maximum	Median	Minimum	Maximum	Median	Minimum	Maximum
1A	+	−	+	10	80.845	28.35	591.01	31.44	2.91	66.11	2.17	0.35	31.05
1B	+	+	+	10	61.96	7.85	591.01	32.61	4.4	53.17	3.26	0.35	22.25
2A	−	+	+	6	100.32	28.76	664.78	32.27	4.4	124.6	0.35	0.35	5.49
2B	−	−	+	4	112.92	45.67	2222.22	4.4	4.4	43.81	1.625	0.35	3.26
3	−	−	−	8	62.395	45.6	389.82	36.56	5.34	65.84	3.82	0.35	28.73

Group	Ampicillin		DIO	*N*	IL-5			IL-6			IL-10		
	Early	Late			Median	Minimum	Maximum	Median	Minimum	Maximum	Median	Minimum	Maximum

1A	+	−	+	10	214.27	2	57.4	37.55	1.1	136.14	155.045	48.06	874.79
1B	+	+	+	10	87.08	32.08	60.14	34.3	1.1	374.97	246.91	70.73	1244.12
2A	−	+	+	6	269.025	55.23	36.38	9.2	1.1	80.87	352.825	146.38	729.41
2B	−	−	+	4	140.725	13.46	4.3	6.7^a^	1.1	12.3	205.345	114.05	1883.25
3	−	−	−	8	164.635	2	4.3	34.05^a^	1.1	51.72	403.235	130.14	642.39

Group	Ampicillin		DIO	*N*	IL-12p70			IL-17			IL-18		
	Early	Late			Median	Minimum	Maximum	Median	Minimum	Maximum	Median	Minimum	Maximum

1A	+	−	+	10	4.3	4.3	57.4	11.24	1.2	25.05	520.83	58.45	1386.39
1B	+	+	+	10	4.3	4.3	60.14	9.21	0.73	28.9	58.45	58.45	1902.05
2A	−	+	+	6	4.3	4.3	36.38	2.425	1.2	20.53	58.45	58.45	1939.3
2B	−	−	+	4	4.3	4.3	4.3	3.485	2.22	4.97	58.45	58.45	4876.61
3	−	−	−	8	4.3	4.3	4.3	8.25	1.2	18.87	58.45	58.45	1939.3

Group	Ampicillin		DIO	*N*	INF*γ*			TNF*α*			GM-CSF		
	Early	Late			Median	Minimum	Maximum	Median	Minimum	Maximum	Median	Minimum	Maximum

1A	+	−	+	10	44.675	5.56	252.73	1.05	1.05	1.05	5.45	5.45	49.69
1B	+	+	+	10	35.04	3.03	444.26	1.05	1.05	1.05	5.45	5.45	24.7
2A	−	+	+	6	35.085	17.95	436.95	1.05	1.05	1.05	5.45	3.74	24.7
2B	−	−	+	4	36.99	24.64	1421.42	1.05	1.05	1.05	5.45	5.45	6.29
3	−	−	−	8	35.915	21.2	333.55	1.05	1.05	1.05	5.45	3.74	24.7

^a^ *P* = 0.039.

**Table 2:** Genes tested by qPCR in ileum tissue of in C57BL/6 mice treated or not treated with Ampicillin from birth to five weeks of age, from 12 to 16 weeks of age, or both.

Gene symbol	Gene	Amplification efficiency (%)		Sequence 5′-3′	Accession number
Actb	Beta-actin	99	F	CCCTAAGGCCAACCGTGAAA	NM_007393.3
			R	CAGCCTGGATGGCTACGTAC	
Alpi	Alkaline phosphatase,	93	F	TCCTAAAGGGGCAGTTGGAA	NM_001081082.1
	Intestinal		R	ACCTGTCTGTCCACGTTGTA	
B2m	Beta-2 microglobulin	100	F	CTGGTGCTTGTCTCACTGAC	NM_009735.3
			R	GGTGGGTGGCGTGAGTATA	
Gusb	Glucuronidase, beta	101	F	AGTATGGAGCAGACGCAATCC	NM_010368.1
			R	ACAGCCTTCTGGTACTCCTCA	
Hp	Haptoglobin	96	F	TATCGCTGCCGACAGTTCTAC	NM_017370.2
			R	CTCTCCAGCGACTGTGTTCA	
Hprt1	Hypoxanthine	97	F	CAGTACAGCCCCAAAATGGTTA	NM_013556.2
	Phosphoribosyltransferase		R	AGTCTGGCCTGTATCCAACA	
Il18	Interleukin 18	98	F	CAAAGAAAGCCGCCTCAAAC	NM_008360.1
			R	GACGCAAGAGTCTTCTGACA	
Il1a	Interleukin 1 alpha	103	F	AGATGGCCAAAGTTCCTGAC	NM_010554.4
			R	AGAGATGGTCAATGGCAGAAC	
Lct	Lactase	92	F	TGTCCTAGCCTACAACCTCAAC	NM_001081078
			R	AGCGGTCTGTAATGGAAGCA	
Muc2	Mucin 2	93	F	TATGCCAGGCCAGGAGTTTA	NM_023566.2
			R	GCAAGGCAGGTCTTTACACA	
Nfkbia	Nuclear factor of kappa, alpha	96	F	GAGCGAGGATGAGGAGAGCTA	NM_010907.2
			R	GGCCTCCAAACACACAGTCA	
Rpl13a	Ribosomal protein L13A	103	F	AGGTTACGGAAACAGGCAGAA	NM_009438.
			R	CAGGAGTCCGTTGGTCTTGA	
Saa	Serum amyloid A	102	F	GAGTCTGGGCTGCTGAGAAA	NM_011314.2
			R	ATGGTGTCCTCGTGTCCTCT	
Tbp	TATA box binding protein	100	F	ACCAGAACAACAGCCTTCCA	NM_013684.3
			R	AAAGATGGGAATTCCAGGAGTCA	
Tgfb1	Transforming growth factor, beta 1	98	F	GCTGCGCTTGCAGAGATTAA	NM_011577.1
			R	GTAACGCCAGGAATTGTTGCTA	
Tlr4	Toll-like receptor 4	98	F	GTTCTTCTCCTGCCTGACAC	NM_021297.2
			R	GCTGAGTTTCTGATCCATGCA	
Tnf	Tumor necrosis factor	95	F	CAAATGGCCTCCCTCTCATCA	NM_013693.2
			R	TGGGCTACAGGCTTGTCAC	
Tnfsf15	Tumor necrosis factor	97	F	GCAAGCCGAGAGCACAC	NM_177371.3
	Superfamily, member 15		R	CCATCCCTAGGTCATGTTCCC	
